# Detection of *mecC-*Positive *Staphylococcus aureus* (CC130-MRSA-XI) in Diseased European Hedgehogs (*Erinaceus europaeus*) in Sweden

**DOI:** 10.1371/journal.pone.0066166

**Published:** 2013-06-12

**Authors:** Stefan Monecke, Dolores Gavier-Widen, Roland Mattsson, Lena Rangstrup-Christensen, Alexandros Lazaris, David C. Coleman, Anna C. Shore, Ralf Ehricht

**Affiliations:** 1 Alere Technologies GmbH, Jena, Germany; 2 Institute for Medical Microbiology and Hygiene, Faculty of Medicine “Carl Gustav Carus”, Technical University of Dresden, Dresden, Germany; 3 Department of Pathology and Wildlife Disease, National Veterinary Institute (SVA), Uppsala, Sweden; 4 Department of Biomedical Sciences and Veterinary Public Health, Swedish University of Agricultural Sciences (SLU), Uppsala, Sweden; 5 Microbiology Research Unit, Dublin Dental University Hospital, University of Dublin, Trinity College Dublin, Dublin, Ireland; 6 Department of Clinical Microbiology, School of Medicine, University of Dublin, Trinity College Dublin, St. James's Hospital, Dublin, Ireland; Catalan Institute for Water Research (ICRA), Spain

## Abstract

Recently, a novel *mec* gene conferring beta-lactam resistance in *Staphylococcus aureus* has been discovered. This gene, *mecC*, is situated on a SCC*mec* XI element that has to date been identified in clonal complexes 49, 130, 425, 599 and 1943. Some of the currently known isolates have been identified from animals. This, and observations of *mecA* alleles that do not confer beta-lactam resistance, indicate that *mec* genes might have a reservoir in *Staphylococcus* species from animals. Thus it is important also to screen wildlife isolates for *mec* genes. Here, we describe *mecC*-positive *Staphylococcus aureus* (ST130-MRSA-XI) and the lesions related to the infection in two diseased free-ranging European hedgehogs (*Erinaceus europaeus*). One was found dead in 2003 in central Sweden, and suffered from *S. aureus* septicaemia. The other one, found on the island of Gotland in the Baltic Sea in 2011, showed a severe dermatitis and was euthanised. ST130-MRSA-XI isolates were isolated from lesions from both hedgehogs and were essentially identical to previously described isolates from humans. Both isolates carried the complete SCC*mec* XI element. They lacked the *lukF-PV/lukS-PV* and *lukM/lukF-P83* genes, but harboured a gene for an exfoliative toxin homologue previously described from *Staphylococcus hyicus*, *Staphylococcus pseudintermedius* and other *S. aureus* of the CC130 lineage. To the best of our knowledge, these are the first reported cases of CC130-MRSA-XI in hedgehogs. Given that one of the samples was taken as early as 2003, this was the earliest detection of this strain and of *mecC* in Sweden. This and several other recent observations suggest that CC130 might be a zoonotic lineage of *S. aureus* and that SCC*mec* XI/*mecC* may have originated from animal pathogens.

## Introduction

Methicillin-resistant *S. aureus* (MRSA) has been known for just over 50 years, and it poses a serious problem for infection prevention and control and antibiotic treatment globally. In MRSA, resistance against almost all beta-lactam compounds in clinical use is caused by the expression of an alternate penicillin-binding protein (PBP2a) that is encoded by the *mecA* gene. It belongs to a family of genes that can be found in various staphylococci [1], 2. *Staphylococcus sciuri* and *Staphylococcus vitulinus* harbour *mecA* alleles that are not associated with beta-lactam resistance [2], [3], [4]. The alleles of *mecA* that are associated with resistance are situated on mobile genetic elements termed Staphylococcal Cassette Chromosome *mec* (SCC*mec*) elements [5] and can be found in *S. aureus* as well as in other staphylococcal species such as *Staphylococcus epidermidis* and *Staphylococcus haemolyticus*. Currently, ten different types of SCC elements harbouring *mecA* as well as a number of subtypes are known from *S. aureus* (see also http://www.sccmec.org/Pages/SCC_TypesEN.html).

A novel *mec* gene type was discovered in 2011 [6], [7]. It is localised on a novel SCC*mec* element designated as type XI [7]. Because of its highly divergent sequence, it cannot be detected by routinely used molecular assays designed to identify *mecA* [7], [8]. This gene, recently renamed *mecC* [1], has been found *S. aureus* belonging to clonal complexes (CCs, as defined by multilocus sequence typing) 49, 130, 425, 599 and 1943. They have been isolated from various animals including cattle, sheep, dogs, cats, a harbour seal (*Phoca vitulina*), a guinea pig, rabbits, rats, and a chaffinch (*Fringilla coelebs*) as well as from humans from Ireland, England, Scotland, Germany, Denmark, Sweden, Norway, France, Switzerland, Belgium and The Netherlands [6], [7], [8], [9], [10], [11], [12], [13], [14], [15], [16], [17], [18], [19], [20]. In Sweden, where this study was performed, *mecC* was previously observed in four out of 537 *S. aureus* isolates recovered from nearly 9,000 cow milk samples [19]. Recently, *mecC* was also found in veterinary *Staphylococcus xylosus* [21].

These observations as well as the observation of *mecA* alleles in animal staphylococci that are not associated with beta-lactam resistance [2], [3] indicate that *mec* genes and their precursors might have a reservoir in animal strains of *Staphylococcus* species. Thus it is important to screen wildlife isolates for *mec* genes.

The aim of the present study was to characterize *mecC*-positive *Staphylococcus aureus* (ST130-MRSA-XI) recovered from two diseased free-ranging European hedgehogs (*Erinaceus europaeus*) from Sweden.

## Materials and Methods

### Animal data and post mortem examination

The two hedgehogs were submitted to the Swedish National Veterinary Institute (SVA) as part of the Swedish wildlife health surveillance program. Hedgehog V583/03 was an adult female found dead in a garden in June 2003 in Bankeryd (Jönköping county, Sweden, 57°51′N 14°07′E). Its body weight was 825 g. The carcass was moderately autolytic.

Hedgehog V5406/11 was an adult male with a body weight of 722 g, found alive in October 2011 on the island Gotland in the Baltic sea, off the East coast of Sweden (57°30′N 18°33′E), and taken care of in a wildlife rehabilitation centre. It had diffuse dermatitis, diarrhoea and difficulties moving, in particular in using the front legs. Due to its poor health it was finally euthanised. Three other hedgehogs with similar clinical signs had been observed in the same area and had died but were not submitted for necropsy.

Routine post mortem examination was performed and selected tissue samples were obtained fresh for bacteriological culture and fixed in 10% neutral buffered formalin for histopathology. The fixed samples were processed and embedded in paraffin wax, sectioned at 5 µm and stained with haematoxylin and eosin (H&E). Selected sections were Gram stained for visualisation of bacteria.

### *S. aureus* isolates

Bacteriological culture was conducted on blood agar base (Difco/Becton Dickinson AB, Stockholm, Sweden) supplemented with 5% citrated equine blood and Bromcresol Purple Lactose Agar (SVA, Uppsala, Sweden). The plates were incubated at 37°C under aerobic conditions and examined after 24 h and 48 h. *S. aureus* was identified by morphological and physiological characteristics. Coagulase-positive *S. aureus* grew in pure culture from the brain and kidneys samples from hedgehog V583/03. An isolate from a kidney was used for further characterisation. Cultures of skin lesions from hedgehog V5406/11 also yielded *S. aureus*.

### Susceptibility tests

Susceptibility tests were performed using the VITEK 2 automated microbiological identification system with the Gram-positive susceptibility panel AST-580 (bioMérieux, Nuertlingen, Germany). In addition, the Clearview ™ (Alere, Cologne, Germany) lateral flow test for the detection of PBP2a was performed according to the protocol provided by the manufacturer using culture material from a MRSA selective growth medium (chromID MRSA, bioMérieux). Minimum inhibitory concentration (MIC) determinations were performed using E-tests (bioMérieux; for methicillin and penicillin) or MIC Test Strips (bestbion dx, Cologne, Germany; for cefoxitin).

### Array procedures

Isolates were characterised using the Alere Technologies StaphyType DNA microarray kit as described previously in detail [22]. The arrays were used to assign isolates to CCs and to identify SCC*mec* associated genes as well as a range of virulence and resistance determinants.

Furthermore, additional probes and primers were introduced to the Alere Technologies microarray and the corresponding master mix, respectively, to identify *mecC* and the *blaZ* allele associated with SCC*mec* XI [6], [7]. Primer and probe sequences are listed in Table 1.

**Table 1:** Novel primer and probe sequences and PCR conditions.

Target	Forward primer	Reverse primer	Hybridisation probe	PCR conditions
*mecC*_out frame_	5′-TGTTGTAGCAATGTTCACAC-3′	5′-CAAGCACTTAATATCAACGC-3′	n.a.	2 min at 95°C/30 cycles (30 sec at 95°C, 30 sec at 55°C, 2 min at 72°C)/5 min at 72°C
*mecC*	5′-ATTAATTGGACCCACATAACC-3′	n.a.	5′-AAAGCCGTGTTTATCCATTGAACGAAG-3′, spotted on the microarray	n.a.
*mecC*	5′-TATAGTTAAATGAAGATCTTTTCCG-3′	n.a.	5′-GGTTTTAAGGTATCCATTGCAAATACTTATGAC-3′, spotted on the microarray	n.a.
*blaZ-M10/0065*	5′-CAGCATTGCGCTACTTATAG-3′	n.a.	5′-AAGCGTTTTGCATATGCTTCCACATTA-3′, spotted on the microarray	n.a.
*etD2*	5′-TCAAGACACCACTAGAAGTC-3′	5′-CGTTTTCAGCTAATCGTGC-3′	n.a.	2 min at 94°C/35 cycles (30 sec at 94°C, 30 sec at 52°C, 30 sec at 72°C)/5 min at 72°C

The identity of the *mec* gene was additionally confirmed using a previously described array that allows the known variants of *mec* from different staphylococcal species to be differentiated [2].

### PCR and sequencing

A PCR for the detection of the SCC*mec*-associated phenol-soluble modulin, PSM-*mec* [23], was performed as previously described [24].

PCRs were also carried out to characterise the SCC*mec* element in detail in both isolates. The complete *mecC* gene was amplified and sequenced using GoTaq DNA polymerase and primers (Table 1) derived from the SCC*mec* XI sequence of an Irish CC130-MRSA-XI isolate (GenBank accession number FR823292.1, [7]). This strain, M10/0061, was also used as a positive control. The entire SCC*mec* XI element including the SCC*mec* XI-associated *ccrA1B3* gene complex was amplified using previously described primers [7]. The sizes of the resulting amplimers obtained were compared to those of M10/0061, from which SCC*mec* XI has been fully sequenced.

The DNA sequence of a novel exfoliative toxin homologue, putatively named *etD2*, that was detected as part of the present study in an Irish CC130 genome sequence [7] as well as in two other CC130 genomes (GenBank accession number AEUQ01000009.1, bases 161312 to 162153 and AEUR01000016.1, bases 44379 to 45221 [25]) was submitted to GenBank under accession number HF563069. The presence of this gene in both isolates was investigated by PCR with primers as listed in Table 1. *S. aureus* reference strains COL (GenBank CP000046) and N315 (GenBank BA000018) were used as negative controls and M10/0061 [7] was used as a positive control. The PCR product had an expected length of ca. 130 bp.

Multilocus sequence typing and *spa* typing were performed on both isolates using previously described protocols [26], [27].The MLST allele sequences were analysed using the MLST website (http://saureus.mlst.net/). The *spa spa* types were assigned using SPATYPEMAPPER software (download at http://www.clondiag.com/fileadmin/Media/Downloads/SPATypeMapper_0_6.zip).

## Results

### Pathology

**Figure 1:**
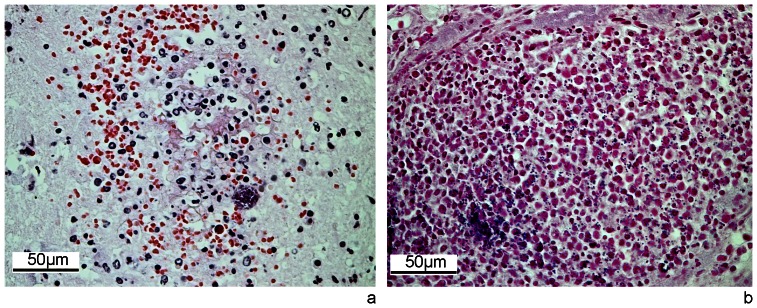
Histological lesions in a hedgehog with MRSA septicaemia (V583/03). a) H&E staining of a brain section showing disruption of the wall of microvasculature with leakage of fibrin and focal haemorrhage, focal infiltrate of inflammatory cells and aggregate of cocci. b) Gram staining of a kidney section showing a focus of necrosis and infiltrate of neutrophils, lymphocytes and macrophages in association to the presence of abundant dispersed or aggregated Gram positive cocci.

Hedgehog V583/03 was in poor physical condition, with depleted fat deposits and pregnant with nine embryonic vesicles. Gross changes included abundant red-brown liquid content in the stomach and intestines, enlarged spleen, pulmonary oedema and hyperaemia and severe hyperaemia of the meninges. Histologically, multiple foci of necrosis and inflammatory infiltration of neutrophils, macrophages and lymphocytes were observed in the cortex and medulla of the kidneys, in the spleen, lungs, meninges and brain parenchyma. In these foci there were numerous Gram-positive cocci, often forming aggregates (Figure 1a). Abundant Gram-positive cocci forming microcolonies were present in the lumen of small blood vessels in multiple organs. The microvasculature of the brain showed disruption of the wall with leakage of fibrin and focal haemorrhages (Figure 1b). In the lungs, eosinophilic bronchitis with nematodes in lumen of bronchi was observed.

**Figure 2:**
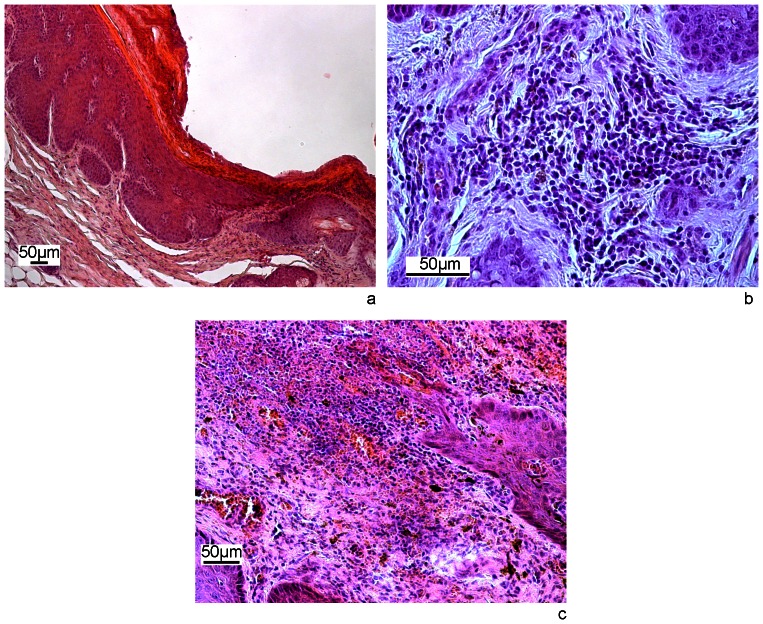
Skin lesions in a hedgehog with MRSA dermatitis (V5406/11). a) H&E staining of an ulceration with necrosis of surface epithelium, thick serocellular crusts and irregular epidermal hyperplasia. b) H&E staining of an inflammatory infiltrate of lymphocytes, macrophages and few neutrophils in the superficial dermis. c) H&E staining of a superficial dermis subjacent to ulceration. Necrosis, cell debris, severe infiltrate of neutrophils and mononuclear cells and haemorrhages.

Hedgehog V5406/11 was in good physical condition but showed a diffuse dermatitis with thickening of the skin and prominent crusts affecting mostly skin areas free of spines. Histologically, skin lesions included ulcerations and formation of serocellular crusts. In the epidermis there was hyperkeratosis, parakeratosis, hyperpigmentation and irregular epidermal hyperplasia (Figure 2a) with formation of rete ridges uneven in length and shape. There was an interface dermatitis with infiltrate of macrophages, lymphocytes and few neutrophils (Figure 2b) and focal perivascular inflammation. Focal necroses and dense infiltration of neutrophils with few mononuclear cells were observed in the superficial dermis (Figure 2c). There was follicular lymphoid hyperplasia in the spleen and eosinophilic bronchitis with presence of nematodes in the lumen of bronchi in the lungs.

### Susceptibility tests of *S. aureus* isolates

Phenotypically, both *S. aureus* isolates were resistant to penicillin and cefoxitin, but the oxacillin MICs as measured by VITEK were low (> = 0.5 µg/mL). However, they were identified by the Vitek software as MRSA based on cefoxitin resistance. Both isolates were susceptible to erythromycin, clindamycin, gentamicin, tobramycin, vancomycin, teicoplanin, tetracyclines, tigecycline, co-trimoxacole, levofloxacin, moxifloxacin, rifampicin, linezolid, mupirocin, fosfomycin and fusidic acid. The oxacillin MICs as determined by agar dilution (E-test) for V583/03 and V5406/11 were 4 µg/mL and 8 µg/mL, respectively, while the cefoxitin MICs were 12 µg/mL and 24 µg/mL, respectively. For both isolates, penicillin MICs were 16 µg/mL.

Both isolates yielded positive signals in the Clearview™ PBP2a lateral flow test.

### Molecular characterisation of *S. aureus* isolates

Both hedgehog isolates belonged to *agr* group III, capsule type 8 and ST130 (MLST alleles *arcC*-6, *aroE*-57, *glpF*-45, *gmk*-2, *pta*-7, *tpi*-58, *yqil*-52). Isolate V5406/11 belonged to *spa* type t843 (repeats: 04-82-17-25-17-25-25-16-17), whereas isolate V583/03 belonged to a similar, but yet not defined *spa* sequence with the repeats 04-17-25-16-17.

Both isolates tested positive for *mecC* and for a SCC*mec* XI-associated beta-lactamase gene *blaZ* by DNA microarray hybridisation as well as, for *mecC*, by PCR and sequencing. The *mecC* gene exhibited 99% DNA sequence identity (nucleotide change of A to C at nucleotide coordinate 1307 within *mecC*) and 100% amino acid sequence identity with *mecC* (GenBank FR821779.1, FR823292.1). In addition, the presence of *ccrA1B3* and the entire SCC*mec* XI element, including the arsenic resistance operon, were confirmed in both isolates using PCR. Both isolates yielded amplicons of the expected size using all primer pairs. The gene encoding the SCC*mec*-associated phenol-soluble modulin PSM-*mec* [23] was absent from both isolates.

Apart from *mecC* and *blaZ*-SCC*mec* XI, no other genes associated with antibiotic resistance were identified.

Full array hybridisation profiles are provided as Table S1. In short, both isolates harboured the *hlg-*locus (*hlgA*, *lukF/S*) and the leukocidin homologue genes *lukD/E*. The Pantone-Valentine leukocidin and the animal-associated leukocidin homologue *lukM/lukF-P83* were absent. The epidermal cell differentiation inhibitor gene *edinB* as well as an exfoliative toxin homologue putatively named “*etD2”* were detected. The *hlb* gene was intact and genes associated with beta-haemolysin converting phages (*sea*, *sep*, *chp*, *sak* and *scn*) were absent. Other enterotoxin genes and the toxic shock toxin gene were also not found. Protease genes *aur*, *splA*, *splB*, *splE*, *sspA*, *sspB* and *sspP* were detected. With regard to adhesion factors, *cna* and *sasG* were absent while *bbp*, *clfA/B coa*, *ebh*, *ebpS*, *eno*, *fib*, *fnbA/B*, *map*, *sdrC/D* and *vwb* were identified.

Hybridisation profiles of both hedgehog isolates were essentially indistinguishable from previously described isolates from Irish patients ([7], see Table S1).

## Discussion

There are very few reports of *S. aureus* and MRSA in hedgehogs. In a study performed in the 1960s in New Zealand, a high prevalence of *S. aureus* (85% of animals tested) was found and a high rate of penicillin resistance (86.3% of isolates tested) was observed. Since these isolates were celbenin (oxacillin) susceptible, the resistance was attributed to a penicillinase [28]. Given the low MICs observed in *mecC*-positive strains as in the present study, its presence cannot safely be ruled out based on observed susceptibility to oxacillin. Thus, it cannot be concluded in retrospect whether these strains were negative for *mecC*, or not. In 2003, MRSA was isolated from a hedgehog suffering from rhinitis in the UK, but no typing data on this isolate were published [29].

To the best of our knowledge, this is the first report of MRSA carrying SCC*mec* XI in hedgehogs. Furthermore, isolate V583/03 recovered in 2003 is, to the best of our knowledge, the earliest known case of a *mecC-*positive *S. aureus* in Sweden. Several recent observations [6], [7], [8], [9], [10], [11], [12], [13], [14], [15], [16], [17], [18], [19], [20] allow the speculation that CC130 might be a zoonotic strain, and that SCC*mec* XI/*mecC* may have originated in animal pathogens.

No host-specific pathotypes for CC130-MRSA-XI can be distinguished, with the hedgehog and human isolates being virtually identical. The animal-specific leukocidin, *lukM/lukF-P83*, was absent from both, hedgehog and human isolates [7], although it has been observed in (methicillin susceptible) sheep and rat isolates of CC130 [7], [25]. The “*etD2*” exfoliative toxin homologue gene has been found in genome sequences of human and animal strains of CC130 [7], [25] as well as in the animal-specific staphylococcal species *Staphylococcus hyicus* (where it was named “*shetb*” ¸ GenBank AB036767.1) and *Staphylococcus pseudintermedius* (“*exi*” or “*expb*”; GenBank AB489850.1 and AB569087.1). A formation of skin blisters and exfoliation upon injection into neonatal mice has been observed [30] and a role in canine pyoderma has been suggested [31] as well as a role in exudative epidermitis of pigs [32]. These observations could suggest a wide host range for staphylococci harbouring “*etD2*”, but more clinical and experimental data are needed to assess the significance of that factor in the different host species.

In humans, CC130-MRSA-XI and other *mecC*-positive strains appear to be rare. In Germany, during the years 2006–2011, less than one out of 1000 MRSA isolates belonged to *mecC*-positive strains [11]. The rate in Denmark is higher, being 1.5% for the period of 2003–2011, and as much as 2.8% in 2011.Their rarity in humans and observations of cases with animal contacts [14] raise the question whether this MRSA strain evolved in animals rather than humans. It is also not yet clear whether domestic or wild animals are a reservoir to *mecC*-positive strains. If they emerged in wild animals such as chaffinches, squirrels, seals, or as in our study, in hedgehogs, the selective pressure(s) that may favour the evolution of MRSA in nature, *i.e*., under low-level exposure to beta-lactam compounds, need to be investigated. However, CC130 MRSA-XI also appears to be uncommon in wildlife, although systematic studies are needed. During Wildtech project activities in Sweden, 46 *S. aureus* isolates from a variety of wild mammal and bird species were identified and genotyped. The two isolates described herein were the only *S. aureus* isolates from hedgehogs, the only *mecC-*positive isolates and the only ones that were assigned to CC130.

The MRSA infection in the hedgehogs described in this report caused severe disease. One of the hedgehogs developed a lethal septicaemia with infection of multiple organs, and MRSA was isolated in abundant growth in pure culture from the two tissues analysed, kidney and brain. In the other case, the hedgehog developed a severe dermatitis. Bacterial shedding and environmental contamination for example through urine and skin purulent exudates is likely to have occurred in these cases. The role of colonised and infected hedgehogs in the epidemiology of *S. aureus/*CC130-MRSA-XI cannot currently be assessed due to lack of information and more studies are required to address this issue.

The presence of *mecA* variants in animal staphylococci [2], [3], [4] as well as of *mecC* in animal isolates of *S. aureus* and *S. xylosus* [21] indicates that bacterial populations from animals might serve as reservoir for precursors of resistance genes, that an emergence of antibiotic resistance in bacteria also takes place in animals and that the rise of drug-resistant pathogens thus can be regarded also as emerging zoonotic disease. Since, hedgehogs in particular often inhabit suburban gardens and parks, people and dogs often come into close contact with them. Therefore, it would be of interest to study the extent and frequency of MRSA infection in hedgehogs and other wildlife as well as the potential risk for humans and pets to contract the infection.

## Supporting Information

Table S1 **Full array hybridisation and PCR results of the hedgehog isolates from this study and, for comparison, of CC130-MRSA-XI isolates from [7].**
(PDF)
