# Supplementary material for: Detection of mecC-Positive Staphylococcus aureus (CC130-MRSA-XI) in Diseased European Hedgehogs (Erinaceus europaeus) in Sweden
Source: PLoS One. 2013 Jun 12;8(6):e66166. doi: 10.1371/journal.pone.0066166 (PMC3680430; doi:10.1371/journal.pone.0066166)
Supplement: Table S1 — Full array hybridisation and PCR results of the hedgehog isolates from this study and, for comparison, of CC130-MRSA-XI isolates from [7]. (PDF) [file pone.0066166.s001.pdf]

Supplemental table S1: Full array hybridisation and PCR results of the hedgehog isolates from this study and, for comparison, of CC130-MRSA-XI isolates from [7].

	Target (Description)	Target (Symbol)	Method [Reference]	DSH_M10/0061	DSH_M10/148	V583/03	V5406/11
BACKGROUND	reference			[7]	[7]	this study	this study
	host species			Human	Human	European Hedgehog	European Hedgehog
	geographic origin			Ireland	Ireland	Sweden (Jönköping)	Sweden (Gotland)
TYPING DATA	spa typing		PCR & Sequencing [27]			undefined 04-17-25-16-17	t843 04-82-17-25-17-25-25-16-17
	MLST typing		PCR & Sequencing [26]	ST130 6-57-45-2-7-58-52	ST1764 6-57-45-2-7-193-52	ST130 6-57-45-2-7-58-52	ST130 6-57-45-2-7-58-52
SPECIES MARKER	Domain 1 of 23SrRNA	rrnD1 (S. aureus)	Array [22]	POS	POS	POS	POS
	glyceraldehyde 3-phosphate dehydrogenase, locus 1	gapA	Array [22]	POS	POS	POS	POS
	katalase A	katA	Array [22]	POS	POS	POS	POS
	coagulase	coA	Array [22]	POS	POS	POS	POS
	thermostable extracellular nuclease	nuc1	Array [22]	POS	POS	POS	POS
	staphylococcal protein A	spa	Array [22]	POS	POS	POS	POS
	IgG-binding protein	sbi	Array [22]	POS	POS	POS	POS
REGULATORY GENES	staphylococcal accessory regulator A	sarA	Array [22]	POS	POS	POS	POS
	histidine protein kinase, sae locus	saeS	Array [22]	POS	POS	POS	POS
	sensor protein	vraS	Array [22]	POS	POS	POS	POS
	accessory gene regulator allele I	agrI (total)	Array [22]	NEG	NEG	NEG	NEG
	accessory gene regulator allele II	agrII (total)	Array [22]	NEG	NEG	NEG	NEG
	accessory gene regulator allele III	agrIII (total)	Array [22]	POS	POS	POS	POS
	accessory gene regulator allele IV	agrIV (total)	Array [22]	NEG	NEG	NEG	NEG
	haemolysin delta	hld	Array [22]	POS	POS	POS	POS
METHICILLIN RESISTANCE AND SCCmec TYPING	alternate penicillin binding protein 2, defining MRSA	mecA	Array [22], Array [2]	NEG	NEG	NEG	NEG
	mecC alternate penicillin binding protein 2 from SCCmec XI	mecC	Array [22, see Table 1], Array [2], PCR, Sequencing	POS	POS	POS	POS
	truncated signal transducer protein MecR1	delta_mecR	Array [22]	NEG	NEG	NEG	NEG
	glycerophosphoryl diester phosphodiesterase, associated with mecA	ugpQ	Array [22]	NEG	NEG	NEG	NEG
	cassette chromosome recombinase genes A/B1	ccrA-1	Array [22]	NEG	NEG	NEG	NEG
		ccrB-1	Array [22]	NEG	AMB	AMB	NEG
	cassette chromosome recombinase gene A1 from SCCmec	ccrA-1 (SCCmec XI)	PCR [7]	POS	POS	POS	POS
	plasmin-sensitive surface protein	plsSCC (COL)	Array [22]	NEG	NEG	NEG	NEG
	hypothetical protein from SCCmec elements	Q9XB68-dcs	Array [22]	NEG	NEG	NEG	NEG
	cassette chromosome recombinase genes A/B2	ccrA-2	Array [22]	NEG	NEG	NEG	NEG
		ccrB-2	Array [22]	NEG	NEG	NEG	NEG
	potassium-translocating ATPase A, chain 2	kdpA-SCC	Array [22]	NEG	NEG	NEG	NEG
	potassium-transporting ATPase B, chain 1	kdpB-SCC	Array [22]	NEG	NEG	NEG	NEG
	potassium-translocating ATPase C, chain 2	kdpC-SCC	Array [22]	NEG	NEG	NEG	NEG
	sensor kinase protein	kdpD-SCC	Array [22]	NEG	NEG	NEG	NEG
	KDP operon transcriptional regulatory protein	kdpE-SCC	Array [22]	NEG	NEG	NEG	NEG
	Phenol soluble modulin associated with SCCmec elements	PSM-mec	PCR [24]	NEG	NEG	NEG	NEG
	methicillinresistance regulatory protein	mecI	Array [22]	NEG	NEG	NEG	NEG
	signal transducer protein MecR1	mecR	Array [22]	NEG	NEG	NEG	NEG
	homolog of xylose repressor, associated with SCCmec elements	xylR	Array [22]	NEG	NEG	AMB	NEG
	cassette chromosome recombinase genes A/B3	ccrA-3	Array [22]	NEG	AMB	NEG	NEG
		ccrB-3	Array [22]	NEG	NEG		NEG
	cassette chromosome recombinase gene B3 from SCCmec XI	ccrB-3 (SCCmec XI)	PCR [7]	POS	POS	POS	POS
	mercury resistance operon	merA	Array [22]	NEG	NEG	NEG	NEG
		merB	Array [22]	NEG	NEG	NEG	NEG
	arsenic resistance operon		PCR [7]	POS	POS	POS	POS
	cassette chromosome recombinase genes "ccrAA" (hypothetical)	ccrAA	Array [22]	NEG	NEG	NEG	NEG
	and ccrC	ccrC (85-2082)	Array [22]	NEG	NEG	NEG	NEG
	cassette chromosome recombinase genes A/B4	ccrA-4	Array [22]	NEG	NEG	NEG	NEG
		ccrB-4	Array [22]	NEG	NEG	NEG	NEG
RESISTANCE : PENICILLINASE	betalactamase	blaZ	Array [22]	NEG	NEG	NEG	NEG
	beta lactamase repressor (inhibitor)	blaI	Array [22]	NEG	NEG	NEG	NEG
	beta lactamase regulatory protein	blaR	Array [22]	NEG	NEG	NEG	NEG
	beta lactamase from SCCmec XI	blaZ-SCCmec XI	Array [22, see Table 1]	POS	POS	POS	POS
RESISTANCE : MLS-ANTIBIOTICS	rRNA methyltransferase, erythromycin/clindamycin resistance	erm(A)	Array [22]	NEG	NEG	NEG	NEG
	erythromycin/clindamycin resistance	erm(B)	Array [22]	NEG	NEG	NEG	NEG
		erm(C)	Array [22]	NEG	NEG	NEG	NEG
	lincosamid-nucleotidyltransferase	lnu(A)	Array [22]	NEG	NEG	NEG	NEG
	energy dependent efflux of erythromycin	msr(A)	Array [22]	NEG	NEG	NEG	NEG
	macrolide efflux protein A	mef(A)	Array [22]	NEG	NEG	NEG	NEG
	probable lysylphosphatidylglycerol synthetase	mph(C)	Array [22]	NEG	NEG	NEG	NEG
	virginiamycin A acetyltransferase	vat(A)	Array [22]	NEG	NEG	NEG	NEG
	acetyltransferase inactivating streptogramin A	vat(B)	Array [22]	NEG	NEG	NEG	NEG
	ATP binding protein, streptogramin A resistance	vga(A)	Array [22]	NEG	NEG	NEG	NEG
		vga(A) (BM 3327)	Array [22]	NEG	NEG	NEG	NEG
	virginiamycin B hydrolase	vgB(A)	Array [22]	NEG	NEG	NEG	NEG
RESISTANCE : AMINOGLYSOSIDES	bifunctional enzyme Aac/Aph, gentamicin resistance	aacA-aphD	Array [22]	NEG	NEG	NEG	NEG
	aminoglycoside adenyltransferase,tobramycin resistance	aadD	Array [22]	NEG	NEG	NEG	NEG
	3'5'aminoglycoside phosphotransferase, neo/ kanamycin resistance	aphA3	Array [22]	NEG	NEG	NEG	NEG
RESISTANCE : MISCELLANEOUS GENES	streptothricin acetyltransferase	sat	Array [22]	NEG	NEG	NEG	NEG
	dihydrofolate reductase type 1	dfrS1	Array [22]	NEG	NEG	NEG	NEG
	fusidic acid resistence	far1	Array [22]	NEG	NEG	NEG	NEG
	hypothetical protein associated with fusidic acid resistance	fusC (Q6GD50)	Array [22]	NEG	NEG	NEG	NEG
	mupirocin resistence protein	mupA	Array [22]	NEG	NEG	NEG	NEG
	tetrazyklin resistance	tet(K)	Array [22]	NEG	NEG	NEG	NEG
		tet(M)	Array [22]	NEG	NEG	NEG	NEG
	chloramphenicol acetyltransferase	cat (total)	Array [22]	NEG	NEG	NEG	NEG
		cat (pC221)	Array [22]	NEG	NEG	NEG	NEG
		cat (pc223)	Array [22]	NEG	NEG	NEG	NEG
		cat (pMC524/pC194)	Array [22]	NEG	NEG	NEG	NEG
		cat (pSBK203R)	Array [22]	NEG	NEG	NEG	NEG
	23S rRNA methyltransferase	cfr	Array [22]	NEG	NEG	NEG	NEG
	chloramphenicol/florfenicol exporter	fexA	Array [22]	NEG	NEG	NEG	NEG
	metallothiol transferase	fosB	Array [22]	NEG	NEG	NEG	NEG
		fosB (plasmid)	Array [22]	NEG	NEG	NEG	NEG
RESISTANCE : EFFLUX SYSTEMS	quaternary ammonium compound resistance protein A	qacA	Array [22]	NEG	NEG	NEG	NEG
	quaternary ammonium compound resistance protein C	qacC (total)	Array [22]	NEG	NEG	NEG	NEG
		qacC (consensus)	Array [22]	NEG	NEG	NEG	NEG
		qacC (equine)	Array [22]	NEG	NEG	NEG	NEG
		qacC (SA5)	Array [22]	NEG	NEG	NEG	NEG
		qacC (Ssap)	Array [22]	NEG	NEG	NEG	NEG
		qacC (ST94)	Array [22]	NEG	NEG	NEG	NEG
	transport/efflux protein	sdrM	Array [22]	POS	POS	POS	POS
RESISTANCE : GLYCOPEPTIDES	vancomycin resistance gene	vanA	Array [22]	NEG	NEG	NEG	NEG
	vancomycin resistance gene from enterococci and Clostridium	vanB	Array [22]	NEG	NEG	NEG	NEG
	teicoplanin resistance gene from enterococci	vanZ	Array [22]	NEG	NEG	NEG	NEG
VIRULENCE : TOX.SCHOCK.TOXIN	toxic shock syndrome toxin 1	tst1 (consensus)	Array [22]	NEG	NEG	NEG	NEG
		tst1 ("human" allele)	Array [22]	NEG	NEG	NEG	NEG
		tst1 ("bovine" allele	Array [22]	NEG	NEG	NEG	NEG
VIRULENCE : ENTEROTOXINS	enterotoxin A	sea	Array [22]	NEG	NEG	NEG	NEG
	enterotoxin A, allele from strain 320E	sea (320E)	Array [22]	NEG	NEG	NEG	NEG
	enterotoxin A, allele from strain N315 = enterotoxin P	sea (N315) / sep	Array [22]	NEG	NEG	NEG	NEG
	enterotoxin B	seb	Array [22]	NEG	NEG	NEG	NEG
	enterotoxin C	sec	Array [22]	NEG	NEG	NEG	NEG
	enterotoxin D	sed	Array [22]	NEG	NEG	NEG	NEG
	enterotoxin E	see	Array [22]	NEG	NEG	NEG	NEG
	enterotoxin G	seg	Array [22]	NEG	NEG	NEG	NEG
	enterotoxin H	seh	Array [22]	NEG	NEG	NEG	NEG
	enterotoxin I	sei	Array [22]	NEG	NEG	NEG	NEG
	enterotoxin J	sej	Array [22]	NEG	NEG	NEG	NEG
	enterotoxin K	sek	Array [22]	NEG	NEG	NEG	NEG
	enterotoxin L	sel	Array [22]	NEG	NEG	NEG	NEG
	enterotoxin M	selm	Array [22]	NEG	NEG	NEG	NEG
	enterotoxin N	seln (consensus)	Array [22]	NEG	NEG	NEG	NEG
		seln (other than RF122)	Array [22]	NEG	NEG	NEG	NEG
	enterotoxin O	selo	Array [22]	NEG	NEG	NEG	NEG
	enterotoxin Q	seq	Array [22]	NEG	NEG	NEG	NEG
	enterotoxin R	ser	Array [22]	NEG	NEG	NEG	NEG
	Enterotoxin U and/or Y	selu/y	Array [22]	NEG	NEG	NEG	NEG
	enterotoxinlike protein ORF CM14	ORF CM14	Array [22]	NEG	NEG	NEG	NEG
VIRULENCE : HLG AND LEUKOCIDINS	haemolysin gamma / leukocidin, component B	lukF	Array [22]	POS	POS	POS	POS
	haemolysin gamma / leukocidin, component C	lukS	Array [22]	POS	POS	POS	POS
		lukS (ST22+ST45)	Array [22]	AMB	POS	AMB	NEG
	haemolysin gamma, component A	hlgA	Array [22]	POS	POS	POS	POS
	Panton Valentine leukocidin F component	lukF-PV	Array [22]	NEG	NEG	NEG	NEG
	Panton Valentine leukocidin S component	lukS-PV	Array [22]	NEG	NEG	NEG	NEG
	F component from hypothetical leukocidin from ruminants	lukF-PV (P83)	Array [22]	NEG	NEG	NEG	NEG
	S component from hypothetical leukocidin from ruminants	lukM	Array [22]	NEG	NEG	NEG	NEG
	leukocidin D component	lukD	Array [22]	POS	NEG	POS	POS
	leukocidin E component	lukE	Array [22]	NEG	NEG	AMB	NEG
	leukocidin/ haemolysin toxin family protein	lukX	Array [22]	POS	POS	POS	POS
	leukocidin/haemolysin toxin family protein	lukY	Array [22]	POS	POS	POS	POS
		lukY (ST30+ST45)	Array [22]	NEG	NEG	NEG	NEG
VIRULENCE : HAEMOLYSINS	putative membrane protein	hl	Array [22]	POS	POS	POS	POS
	haemolysin alpha	hla	Array [22]	POS	POS	POS	POS
	putative membrane protein	hlIII (consensus)	Array [22]	POS	POS	POS	POS
		hlIII (other than RF122)	Array [22]	NEG	NEG	NEG	NEG
	haemolysin beta	hlb_probe 1	Array [22]	POS	POS	POS	POS
		hlb_probe 2	Array [22]	POS	POS	POS	POS
		hlb_probe 3	Array [22]	POS	POS	POS	POS
		un-disrupted hlb	Array [22]	POS	POS	POS	POS
VIRULENCE : HLB-CONV PHAGES	staphylokinase	sak	Array [22]	NEG	NEG	NEG	NEG
	chemotaxisinhibiting protein (CHIPS)	chp	Array [22]	NEG	NEG	NEG	NEG
	staphylococcal complement inhibitor	scn	Array [22]	NEG	NEG	NEG	NEG
VIRULENCE : EXFOL.TOXINS	exfoliative toxin serotype A	etA	Array [22]	NEG	NEG	NEG	NEG
	exfoliative toxin serotype B	etB	Array [22]	NEG	NEG	NEG	NEG
	exfoliative toxin D	etD	Array [22]	NEG	NEG	NEG	NEG
	putative exfoliative toxin homologue	etD2	PCR [see table 1]	POS		POS	POS
VIRULENCE : EPIDERMAL CELL DIFF. INHIBITOR	epidermal cell differentiation inhibitor	edinA	Array [22]	NEG	NEG	NEG	NEG
	epidermal cell differentiation inhibitor B	edinB	Array [22]	POS	POS	POS	POS
	epidermal cell differentiation inhibitor C	edinC	Array [22]	NEG	NEG	NEG	NEG
VIRULENCE : ACME LOCUS	ACME locus	ACME (total)	Array [22]	NEG	NEG	NEG	NEG
	ACME locus: arginine dihydrolase	arcA-SCC	Array [22]	NEG	NEG	NEG	NEG
	ACME locus: ornithincarbamoyltransferase	arcB-SCC	Array [22]	NEG	NEG	NEG	NEG
	ACME locus: carbamatkinase	arcC-SCC	Array [22]	NEG	NEG	NEG	NEG
	ACME locus: arginine/ornithineantiporter	arcD-SCC	Array [22]	NEG	NEG	NEG	NEG

	Target (Description)	Target (Symbol)	Method [Reference]	DSH_M10/0061	DSH_M10/148	V583/03	V5406/11
**VIRULENCE : PROTEASES**	aureolysin	*aur (consensus)*	Array [22]	POS	POS	POS	POS
		aur (other than MRSA252)	Array [22]	POS	POS	POS	POS
		aur (MRSA252)	Array [22]	NEG	NEG	NEG	NEG
	serin protease A	*splA*	Array [22]	POS	NEG	POS	POS
	serin protease B	*splB*	Array [22]	POS	NEG	POS	POS
	serin protease E	*splE*	Array [22]	POS	NEG	POS	POS
	glutamylendopeptidase	*sspA*	Array [22]	POS	POS	POS	POS
	staphopain B, protease	*sspB*	Array [22]	POS	POS	POS	POS
	staphopain A (staphylopain A), protease	*sspP (consensus)*	Array [22]	POS	POS	POS	POS
		sspP (other than ST93,	Array [22]	POS	POS	POS	POS
**VIRULENCE : STAPHYLOCOCCAL SUPERANTIGEN / ENTEROTOXIN-LIKE GENES (SET/SSL)**	staphyl. exotoxinlike protein	*setC (=selX)*	Array [22]	POS	POS	POS	POS
	staphylococcal superantigenlike protein 1	*ssl01/set6 (COL)*	Array [22]	NEG	NEG	NEG	NEG
		ssl01/set6 (Mu50+N315)	Array [22]	POS	POS	POS	POS
		ssl01/set6 (MW2+MSSA476)	Array [22]	NEG	NEG	NEG	NEG
		ssl01/set6 (MRSA252)	Array [22]	NEG	NEG	NEG	NEG
		ssl01/set6 (RF122)	Array [22]	NEG	NEG		NEG
		ssl01/set6 (other alleles)	Array [22]	NEG	NEG	NEG	NEG
	staphylococcal superantigenlike protein 2	*ssl02/set7*	Array [22]	POS	POS	POS	POS
		ssl02/set7 (MRSA252)	Array [22]	NEG	NEG	NEG	NEG
	staphylococcal superantigenlike protein 3	*ssl03/set8_probe 1*	Array [22]	POS	POS	POS	POS
		ssl03/set8_probe 2	Array [22]	POS	POS	POS	POS
		ssl03/set8 (MRSA252, SAR0424)	Array [22]	NEG	NEG	NEG	NEG
	staphylococcal superantigenlike protein 4	*ssl04/set9*	Array [22]	POS	POS	POS	POS
		ssl04/set9 (MRSA252, SAR0425)	Array [22]	NEG	AMB	NEG	NEG
	staphylococcal superantigenlike protein 5	*ssl05/set3_probe 1*	Array [22]	NEG	NEG	NEG	NEG
		ssl05/set3 (RF122, probe-611)	Array [22]	POS	POS	AMB	POS
		ssl05/set3_probe 2 (612)	Array [22]	AMB	AMB	POS	AMB
		ssl05/set3 (MRSA252)	Array [22]	NEG	NEG	NEG	NEG
	staphylococcal superantigenlike protein 6	*ssl06/set21*	Array [22]	NEG	NEG	NEG	NEG
		ssl06 (NCTC8325+MW2)	Array [22]	NEG	NEG	NEG	NEG
	staphylococcal superantigenlike protein 7	*ssl07/set1*	Array [22]	POS	POS	POS	POS
		ssl07/set1 (MRSA252)	Array [22]	AMB	AMB	AMB	AMB
		ssl07/set1 (AF188836)	Array [22]	NEG	AMB	AMB	NEG
	staphylococcal superantigenlike protein 8	*ssl08/set12_probe 1*	Array [22]	AMB	POS	POS	AMB
		ssl08/set12_probe 2	Array [22]	POS	POS	POS	AMB
	staphylococcal superantigenlike protein 9	*ssl09/set5_probe 1*	Array [22]	POS	POS	POS	POS
		ssl09/set5_probe 2	Array [22]	POS	POS	POS	POS
		ssl09/set5 (MRSA252)	Array [22]	NEG	NEG	NEG	NEG
	staphylococcal superantigenlike protein 10	*ssl10/set4*	Array [22]	POS	AMB	POS	AMB
		ssl10 (RF122)	Array [22]	POS	POS	AMB	POS
		ssl10/set4 (MRSA252)	Array [22]	NEG	AMB	NEG	NEG
	staphylococcal superantigenelike protein 11	*ssl11/set2 (COL)*	Array [22]	NEG	NEG	NEG	NEG
		ssl11+set2(Mu50+N315)	Array [22]	NEG	NEG	NEG	NEG
		ssl11+set2(MW2+MSSA476)	Array [22]	NEG	NEG	NEG	NEG
		ssl11/set2 (MRSA252)	Array [22]	NEG	NEG	NEG	NEG
	staphylococcal exotoxinlike protein, second locus	*setB3*	Array [22]	POS	POS	POS	POS
		setB3 (MRSA252)	Array [22]	NEG	NEG	NEG	NEG
		setB2	Array [22]	POS	POS	POS	POS
		setB2 (MRSA252)	Array [22]	NEG	NEG	NEG	NEG
		setB1	Array [22]	POS	POS	POS	POS
**CAPSULE- AND BIOFILM-ASSOCIATED GENES**	capsule type 1	*cap 1 (total)*	Array [22]	NEG	NEG	NEG	NEG
	capsule type 5	*cap 5 (total)*	Array [22]	NEG	NEG	NEG	NEG
	capsule type 8	*cap 8 (total)*	Array [22]	POS	POS	POS	POS
	intercellular adhesion protein A	*icaA*	Array [22]	POS	POS	POS	POS
	intercellular adhesion protein C	*icaC*	Array [22]	POS	POS	POS	POS
	biofilm PIA synthesis protein D	*icaD*	Array [22]	POS	POS	POS	POS
	surface protein involved in biofilm formation	*bap*	Array [22]	NEG	NEG	NEG	NEG
**ADHAESION FACTORS / GENES ENCODING MICROBIAL SURFACE COMPONENTS RECOGNIZING ADHESIVE MATRIX MOLECULES (MSCRAMM GENES)**	bone sialoproteinbinding protein	*bbp (total)*	Array [22]	POS	POS	POS	POS
		bbp (consensus)	Array [22]	POS	POS	POS	POS
		bbp (COL+MW2)	Array [22]	NEG	NEG	NEG	NEG
		bbp (MRSA252)	Array [22]	NEG	NEG	NEG	NEG
		bbp (Mu50)	Array [22]	AMB	POS	POS	NEG
		bbp (RF122)	Array [22]	NEG	NEG	NEG	NEG
		bbp (ST45)	Array [22]	NEG	NEG	NEG	NEG
	clumping factor A	*clfA (total)*	Array [22]	POS	POS	POS	POS
		clfA (consensus)	Array [22]	POS	POS	POS	POS
		clfA (COL+RF122)	Array [22]	POS	POS	POS	POS
		clfA (MRSA252)	Array [22]	AMB	AMB	AMB	NEG
		clfA (Mu50+MW2)	Array [22]	AMB	AMB	AMB	AMB
	clumping factor B	*clfB (total)*	Array [22]	POS	POS	POS	POS
		clfB (consensus)	Array [22]	POS	POS	POS	POS
		clfB (COL+Mu50)	Array [22]	NEG	NEG	NEG	NEG
		clfB (MW2)	Array [22]	AMB	AMB	AMB	AMB
		clfB (RF122)	Array [22]	POS	POS	POS	POS
	collagen binding adhesin	*cna*	Array [22]	NEG	NEG	AMB	NEG
	cell wall associated fibronectinbinding protein	*ebh (consensus)*	Array [22]	POS	POS	POS	POS
	cell surface elastin binding protein	*ebpS (total)*	Array [22]	POS	POS	POS	POS
		ebpS_probe 612	Array [22]	POS	POS	POS	POS
		ebpS_probe 614	Array [22]	POS	POS	POS	POS
		ebpS (01-1111)	Array [22]	NEG	NEG	NEG	NEG
		ebpS (COL)	Array [22]	NEG	NEG	NEG	NEG
	enolase	*eno*	Array [22]	POS	POS	POS	POS
	fibrinogen binding protein (19 kDa)	*fib*	Array [22]	POS	POS	POS	POS
		fib (MRSA252)	Array [22]	NEG	NEG	NEG	NEG
	fibronectinbinding protein A	*fnbA (total)*	Array [22]	POS	POS	POS	POS
		fnbA (consensus)	Array [22]	POS	POS	POS	POS
		fnbA (COL)	Array [22]	NEG	NEG	NEG	NEG
		fnbA (MRSA252)	Array [22]	NEG	NEG	NEG	NEG
		fnbA (Mu50+MW2)	Array [22]	NEG	NEG	NEG	NEG
		fnbA (RF122)	Array [22]	NEG	NEG	NEG	NEG
	fibronectinbinding protein B	*fnbB (total)*	Array [22]	POS	POS	POS	POS
		fnbB (COL)	Array [22]	NEG	NEG	NEG	NEG
		fnbB (COL+Mu50+MW2)	Array [22]	POS	POS	POS	POS
		fnbB (Mu50)	Array [22]	NEG	NEG	NEG	NEG
		fnbB (MW2)	Array [22]	NEG	NEG	NEG	NEG
		fnbB (ST15)	Array [22]	NEG	NEG	NEG	NEG
		fnbB (ST45-2)	Array [22]	NEG	NEG	NEG	NEG
	Major histocompatibility complex class II analog protein (=Extracellular adherence protein, eap)	*map (total)*	Array [22]	POS	POS	POS	POS
		map (COL)	Array [22]	POS	POS	POS	POS
		map (MRSA252)	Array [22]	NEG	NEG	NEG	NEG
		map (Mu50+MW2)	Array [22]	NEG	POS	POS	NEG
	Staphylococcus aureus surface protein G	*sasG (total)*	Array [22]	NEG	NEG	NEG	NEG
		sasG (COL+Mu50)	Array [22]	NEG	NEG	NEG	NEG
		sasG (MW2)	Array [22]	NEG	NEG	NEG	NEG
		sasG (other than MRSA252+RF122)	Array [22]	NEG	NEG	NEG	NEG
	SerAsp rich fibrinogen/bone sialoprotein binding protein C	*sdrC (total)*	Array [22]	POS	POS	POS	POS
		sdrC (consensus)	Array [22]	POS	POS	POS	POS
		sdrC (B1)	Array [22]	NEG	NEG	NEG	NEG
		sdrC (COL)	Array [22]	POS	POS	POS	POS
		sdrC (Mu50)	Array [22]	NEG	NEG	NEG	NEG
		sdrC (MW2+MRSA252+RF122)	Array [22]	NEG	NEG	NEG	NEG
		sdrC (other than MRSA252+RF122)	Array [22]	NEG	NEG	NEG	NEG
	SerAsp rich fibrinogen/bone sialoprotein binding protein D	*sdrD (total)*	Array [22]	POS	POS	POS	POS
		sdrD (consensus)	Array [22]	POS	POS	POS	POS
		sdrD (COL+MW2)	Array [22]	NEG	NEG	NEG	NEG
		sdrD (Mu50)	Array [22]	NEG	AMB	NEG	NEG
		sdrD (other)	Array [22]	NEG	NEG	NEG	NEG
	van Willebrand factor binding protein	*vwb (total)*	Array [22]	POS	POS	POS	POS
		vwb (consensus)	Array [22]	POS	POS	POS	POS
		vwb (COL+MW2)	Array [22]	NEG	NEG	NEG	NEG
		vwb (MRSA252)	Array [22]	NEG	NEG	NEG	NEG
		vwb (Mu50)	Array [22]	NEG	NEG	NEG	NEG
		vwb (RF122)	Array [22]	NEG	NEG	NEG	NEG
**TYPE I RESTRICTION-MODIFICATION SYSTEM,**	type I site-specific deoxyribonuclease subunit, 1st locus	*hsdS1 (RF122)*	Array [22]	NEG	NEG	NEG	NEG
	type I site-specific deoxyribonuclease subunit, 2nd locus	*hsdS2 (Mu50+N315+COL+USA300+NCTC8325)*	Array [22]	NEG	NEG	NEG	NEG
		hsdS2 (MW2+MSSA476)	Array [22]	NEG	NEG	AMB	NEG
		hsdS2 (RF122)	Array [22]	POS	POS	POS	POS
		hsdS2 (MRSA252)	Array [22]	NEG	NEG	NEG	NEG
	type I site-specific deoxyribonuclease subunit, 3rd locus	*hsdS3 (all other than RF122+ MRSA252)*	Array [22]	NEG	POS	NEG	NEG
		hsdS3 (COL+USA300+NCTC8325+MW2+MSSA476+RF122)	Array [22]	POS	NEG	POS	POS
		hsdS3 (Mu50+N315)	Array [22]	NEG	NEG	NEG	NEG
		hsdS3 (CC51+ MRSA252)	Array [22]	NEG	NEG	NEG	NEG
		hsdS3 (MRSA252)	Array [22]	NEG	NEG	NEG	NEG
	type I site-specific deoxyribonuclease subunit, unknown locus	*hsdSx (CC25)*	Array [22]	POS	POS	POS	POS
		hsdSx (CC15)	Array [22]	NEG	AMB	NEG	NEG
		hsdSx (etd)	Array [22]	POS	POS	POS	POS
**HYALURONATE LYASE**	hyaluronate lyase, first / second locus	*hysA1 (MRSA252)*	Array [22]	NEG	NEG	NEG	NEG
		hysA1 (MRSA252+RF122) and/or hysA2 (consensus)	Array [22]	POS	POS	POS	POS
		hysA1 (MRSA252+RF122) and/or hysA2 (COL+USA300)	Array [22]	NEG	NEG	NEG	NEG
	hyaluronate lyase, second locus	*hysA2 (all other than MRSA252)*	Array [22]	NEG	NEG	NEG	NEG
		hysA2 (COL+USA300+NCTC8325)	Array [22]	NEG	NEG	NEG	NEG
		hysA2 (all other than COL+USA300+NCTC8325)	Array [22]	POS	POS	POS	POS
		hysA2 (all other than COL+USA300+NCTC8325)	Array [22]	POS	POS	POS	POS
		hysA2 (MRSA252)	Array [22]	NEG	NEG	NEG	NEG
**MISCELLANEOUS GENES**	hypothetical protein, located next to serine protease operon	*Q2FXC0*	Array [22]	NEG	NEG	NEG	NEG
	unspecific efflux/transporter	*Q2YUB3*	Array [22]	NEG	AMB	NEG	NEG
	hypothetical protein	*Q7A4X2*	Array [22]	NEG	NEG	NEG	NEG
	immunodominant antigen B	*isaB*	Array [22]	POS	POS	POS	POS
		isaB (MRSA252)	Array [22]	AMB	AMB	AMB	AMB
	defensin resistance protein	*mprF (COL+MW2)*	Array [22]	POS	POS	POS	POS
		mprF (Mu50+MRSA252)	Array [22]	AMB	AMB	AMB	AMB
	transferrin-binding protein	*isdA (consensus)*	Array [22]	POS	POS	POS	POS
		isdA (MRSA252)	Array [22]	NEG	AMB	AMB	NEG
		isdA (other than MRSA252 ,	Array [22]	POS	POS	POS	POS
	hypothetical protein, similar to integral membrane protein LmrP	*lmrP (other than RF122)*	Array [22]	NEG	NEG	NEG	NEG
		lmrP (RF122)	Array [22]	POS	POS	POS	POS
